# Molecular dissection of Xinong 511 spike rachis response to *Fusarium* head blight infection

**DOI:** 10.1007/s44154-025-00240-x

**Published:** 2025-07-23

**Authors:** Xiaoying Yang, Maoru Xu, Guangyi Wang, Xiaofang Cheng, Zhengkai Feng, Xiaoqi Zhao, Tingdong Li, Pingchuan Deng, Changyou Wang, Xinlun Liu, Jixin Zhao, Chunhuan Chen, Wanquan Ji

**Affiliations:** 1 College of Agronomy, Northwest A&F University, Yangling, 712100 China; 2 State Key Laboratory of Crop Stress Biology for Arid Areas, Yangling, 712100 China

**Keywords:** *Fusarium* head blight, *Triticum aestivum *L., Disease resistance, Phenylpropanoid pathway, WGCNA

## Abstract

*Fusarium* head blight (FHB, also known as wheat scab or ear blight), caused primarily by the *Fusarium graminearum*, is a worldwide disease of wheat (*Triticum aestivum *L.). Studying the pathogen expansion patterns and molecular mechanisms of disease resistance in resistant wheat varieties is crucial for advancing wheat disease management strategies. Here, we found a significant difference between two wheat cultivars with different resistances, and it was revealed that they exhibited divergent pathogen infestation process. The susceptible cultivar showed extensive pathogen in the spike rachis, while resistant varieties only had limited pathogen spread and colonization. Meanwhile, wheat resistance to FHB was positively correlated with transcriptional reprogramming in the early stages, with higher expression of genes responding to plant defense related genes and phenylpropanoid pathway genes in the early stages of disease resistant variety. Weighted gene co-expression network analysis (WGCNA) of differential expression genes (DEGs) analysis led to the construction of a network modules associated with resistance genes, and an important role of heavy metal-associated (*HMA*) domain protein in plant defense was identified in the tan module. RNA-induced gene silencing preliminarily identified two key genes that resistance to FHB in wheat: a cytochrome P450 (*CYP*) gene involved in the flavonoid biosynthesis within the phenylpropanoid pathway and *HMA* gene. This study provides an in-depth analysis of the infection mechanisms of wheat by *F. graminearum* and elucidates the key molecular mechanisms involved, while being useful for advancing the breeding of wheat varieties resistant to FHB.

**Supplementary Information:**

The online version contains supplementary material available at 10.1007/s44154-025-00240-x.

## Introduction

As one of the “big three” cereals, wheat plays an important role in the human diet by providing us with several key nutrients (Shewry 2009). However, climate change and certain cropping practices have introduced multiple biotic and abiotic challenges in wheat production (Wang et al. 2020). Among these challenges, FHB, a devastating disease caused by *Fusarium graminearum* Schwabe (*Gibberella zeae* (Schw.) Perch), affects not only wheat but also a wide range of crops, including barley and maize (Dweba et al. 2017). FHB causes enormous yield losses and deteriorates grain quality (Huang et al. 2024; Kazan et al. 2012). Control strategies for *F. graminearum* rely heavily on the breeding of resistant wheat varieties and using chemical fungicides (Drakopoulos et al. 2020). Therefore, a comprehensive understanding of the molecular mechanisms underlying the pathogenicity of *F. graminearum* is crucial for developing effective strategies for disease management.

During the flowering period of wheat, spores of *F. graminearum* land on flowering spike and enter the florets through the gap between the palea and lemma. Initially, the fungus grows intercellularly and asymptomatically, then penetrates through the xylem to the rachis and begins to invade the cells (Trail 2009). Once the fungus has colonized the tissue, necrosis begins to occur, and the phenotypic characteristics of the disease become apparent. The types of resistance to FHB are further differentiated by the different manifestations of the plant, including resistance to initial infection (type I), disease spread (type II), toxin accumulation (type III), kernel infection (type IV), and yield loss (type V) (Dweba et al. 2017; Mesterházy 1995). Due to the complexity of the resistance mechanisms of wheat against *F. graminearum*, our understanding of these mechanisms was very limited, which has hindered the progress in breeding wheat resistant to FHB and failed to meet the practical demands. The use of resistance genes or varieties is considered an economical and environmentally safe method to control plant diseases in crop breeding programs (Zhang et al. 2023).

To defend against diverse microbial pathogens, plants have evolved a robust immune system (Dodds and Rathjen 2010). Numerous studies have found that phenylpropanoid compounds can indeed contribute effectively to plant resistance (Dong and Lin 2021; Naoumkina et al. 2010). Phenylalanine ammonia lyase (PAL) is the gateway enzyme of the general phenylpropanoid pathway, directing the catalysis of the formation of trans-cinnamic acid from phenylalanine (Zhang and Liu 2015). This enzyme guides the metabolic flow into various branches of the phenylpropanoid pathway, leading to the synthesis pathways of secondary metabolites such as flavonoids, anthocyanins, lignin, which are closely associated with plant defense responses (Ferrer et al. 2008; Menden et al. 2007; Ranjan et al. 2019). Studies have revealed that in wheat, the silencing genes in this pathway increased susceptibility to *Blumeria graminis* f. sp. *tritici* (*Bgh*) infection (Bhuiyan et al. 2008). In highly resistant of stem rust fungus (*Puccinia graminis* f.sp. *tritici*) wheat cultivars, fungal attack is stopped by a hypersensitive response of penetrated host cells. Analysis of the biochemical composition of this defense showed an increase in lignin content after elicitor treatment (Menden et al. 2007). CYPs belong to a class of oxidoreductases enzyme and also involved in protecting plants from harsh environmental conditions by enhancing the activity of compounds (e.g., flavonoids) with increased antioxidant activity (Pandian et al. 2020). In the wheat ‘Ning7840’, the genes encoding CYPs and chitinase were found to be upregulated following infection by the pathogen, indicating their active response to *F. graminearum* infection (Kong et al. 2005). In summary, plants enhance their immune system to fight against a variety of pathogens through complex metabolic pathways and the synthesis of diverse compounds.

Many toxic secondary metabolites produced by plant phytopathogenic fungi can destroy host cells (Nesic et al. 2014), and thus detoxification of mycotoxins can be effective in reducing the toxic effects of pathogenic fungi on the host (Nesic et al. 2014; Wang et al. 2021). This allows plants to maintain their innate immunity against pathogens invasion and increase their resistance to diseases caused by toxin producers. For example, the UDP-glucosyltransferase (UGT) gene improves resistance to FHB resistance as type II in wheat (Li et al. 2015). UGT can conjugate deoxynivalenol (DON) into nontoxic DON-3-O-glucoside (D3G), thus reducing toxicity to plant cells (Bethke et al. 2023). The Arabidopsis P4 ATPases *AtALA1* and *AtALA7* were responsible for cellular detoxification of mycotoxins, and their overexpression significantly increases the resistance of transgenic plants to *F. graminearum* and *Verticillium dahliae*, respectively (Wang et al. 2021). The role of ions in combating plants pathogens has long been overlooked, mainly because metal ion research has been predominantly focused on metal contamination in agricultural soils. Both excessive accumulation and deficiency of metal ions can affect the activity of metal cation-dependent enzymes, thereby altering the homeostasis of plant metabolic pathways (Clemens and Ma 2016). Alterations in plant metabolic pathways can significantly affect plant disease resistance. For instance, in *Vitis vinifera* infected with *plasmopara viticola,* a dramatic redistribution of mineral elements has been observed in susceptible cultivars, while microelements play a role in secondary metabolism and reactive oxygen species in resistant cultivar (Cesco et al. 2020). The rice heavy-metal transporter *OsNRAMP1* plays an important role in plant immunity by modulating metal ions and ROS homoeostasis (Chu et al. 2022). *OsCNGC9*, encoding a cyclic nucleotide-gated channel protein, positively regulates resistance to rice blast disease by promoting PAMPs-triggered ROS bursts and the induction of PTI-related defense gene expression (Wang et al. 2019). Host plants employ various strategies to regulate metabolic homeostasis by modulating the expression of metal ion-related proteins to enhance resistance to pathogens.

Our laboratory has developed the wheat‐*Thinopyrum ponticum* introgression variety named Xinong 511 (XN511) with resistance to FHB through distant hybridization (Yang et al. 2024). This cultivar is known for its high quality, strong tillering ability, and robust resistance to lodging and spring cold. XN511 has become a leading cultivar in the Yellow and Huai River Valleys of China, with an area of more than 2 million hectares and an average yield of 7996.5 kg/hm^2^ (http://202.127.42.145/bigdataNew/). To investigate the molecular mechanisms of XN511 under *F. graminearum* infection, we selected the susceptible wheat variety Aikang 58 (AK58) as a reference and conducted a comparative analysis of pathogen colonization and expansion between the two materials. Since its release in 2005, AK58 has been a leading elite winter wheat cultivar in the Yellow and Huai River Valley region, exhibits strong resistance to lodging and increased tolerance to multiple abiotic stresses (Hao et al. 2020; Jia et al. 2021, 2023). In this study, we described a detailed microscopic investigation of the entire colonization of the *F. graminearum* on both susceptible and resistant wheat ears. Our investigation traced the fungal progression from the initially inoculated florets into the rachis nodes. Samples from the two cultivars and the recombinant inbred lines (RILs) derived from their cross were collected during the infection process to analyze their transcriptional dynamics. Ultimately, we identified two resistance genes and preliminarily their functions through RNA-induced gene silencing. This study contributes significantly to understanding the resistance mechanisms against FHB in wheat.

## Results

### Rachis determined the plant defense against FHB at wheat

**Fig. 1:**
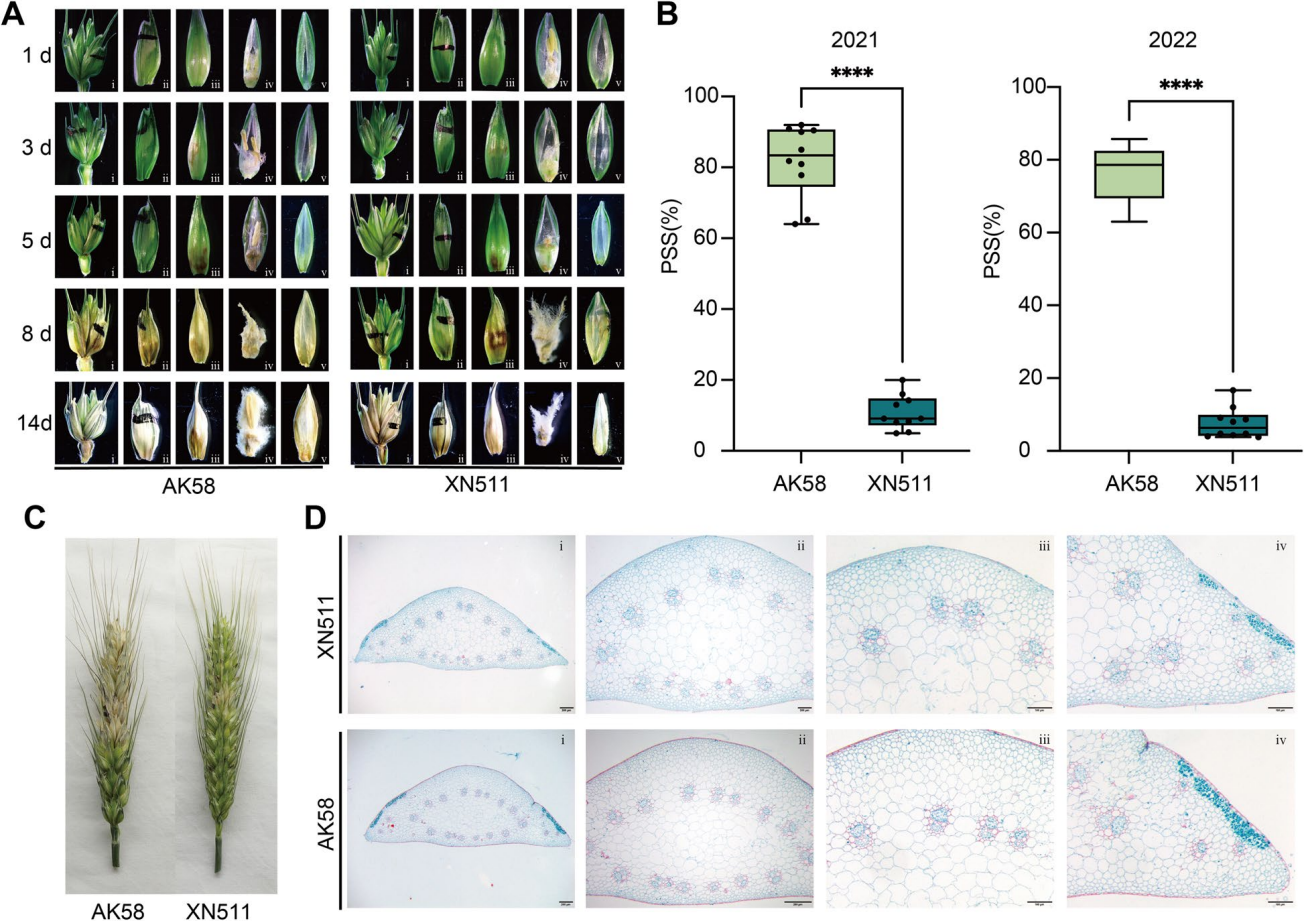
*Fusarium* head blight resistance in Xinong 511 and Aikang 58. **A** Morphological development of inoculated spikelet tissue from 1 to 14d after *Fusarium-*infected in AK58 (left) and XN511 (right) (*n* = 5). Each floret was separated: i, indicating the inoculated spikelet; ii, the glume of the inoculated spikelet; iii, the lemma of the inoculated spikelet; iv, the grain of the inoculated spikelet; v, the palea of the inoculated spikelet. In the PH1 infected ear, the kernel remained in the floral cavity but had not developed post inoculation and appeared shriveled at 8 dpi. **B** Percentage of symptomatic spikelet (PSS) for XN511 and AK58 in 2021 and 2022. Data are presented as means ± standard deviation (SD) (*n* = 10, ****P < 0.0001; Student’s *t*-test). **C** Spike inoculation with F. *graminearum* phenotypes in XN511 and AK58. **D** Microscopic observation of rachis of XN511 and AK58. Sections were stained by van gieson stain. The scale bar is located at the bottom right of the images. The lignified cell walls are red, while the cellulose cell walls are green

**Fig. 2:**
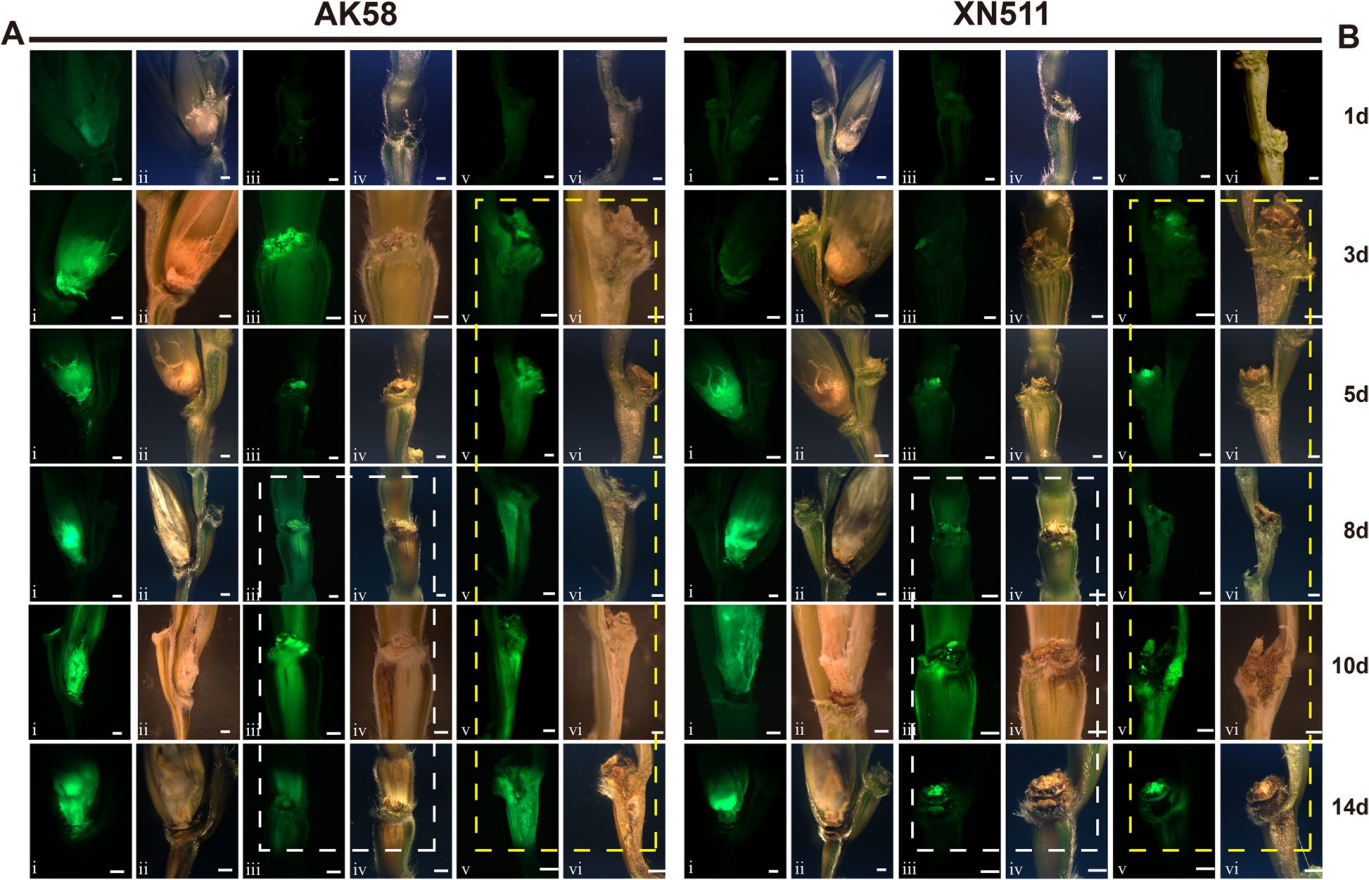
Different phenotypes of rachis of resistant and susceptible plants under *Fusarium graminearum* infestation. The hyphae distribution in the susceptible (**A**) and resistant (**B**) wheat florets and rachis tissue were revealed by fluorescence scanning microscopy at different time after *F. graminearum* infection at 1, 3, 5, 8, 10 and 14 dpi. Photographs were taken in the dark field for the detection of green fluorescence (inoculation site palea, images marked i; inoculation site rachis, images marked iii and inoculation site rachis cross section, images marked v) and in the bright field for the evaluation of disease phenotype (inoculation site palea, images marked ii; inoculation site rachis, images marked iv and inoculation site rachis cross section, images marked vi). The white dashed boxes represent the differential phenotypes of the spike rachis in the two materials after *F. graminearum* infection, and the yellow dashed boxes represent the differential phenotypes of the inoculation site rachis cross section. The treatment stages are listed on the right of each panel. Scale bars are shown in the lower right corner of each image

**Fig. 3:**
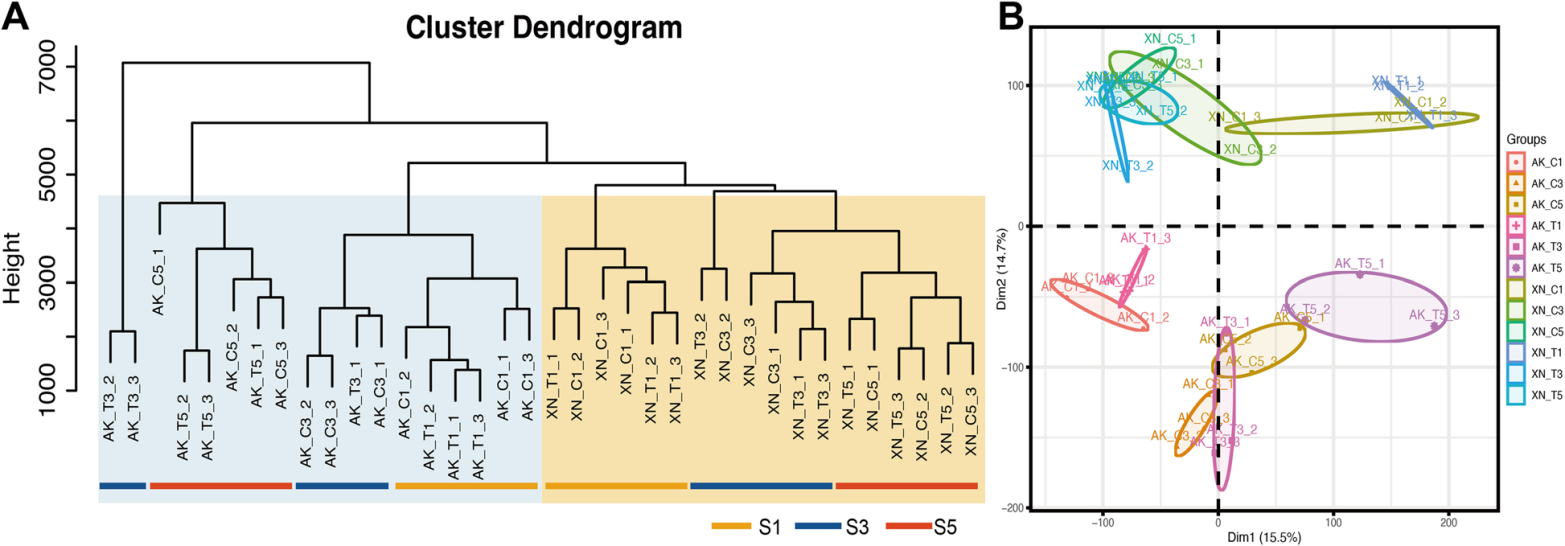
Transcriptome relationships among three stages of resistant and susceptible materials. **A** The clustering tree diagram shows different clustering groups. **B** Principal component analysis of genes identified in all samples. S1 represents 1 day after inoculation and mock inoculation (T1 and C1), S3 represents 3 days after (T3 and C3), and S5 represents 5 days after (T5 and C5)

**Fig. 4:**
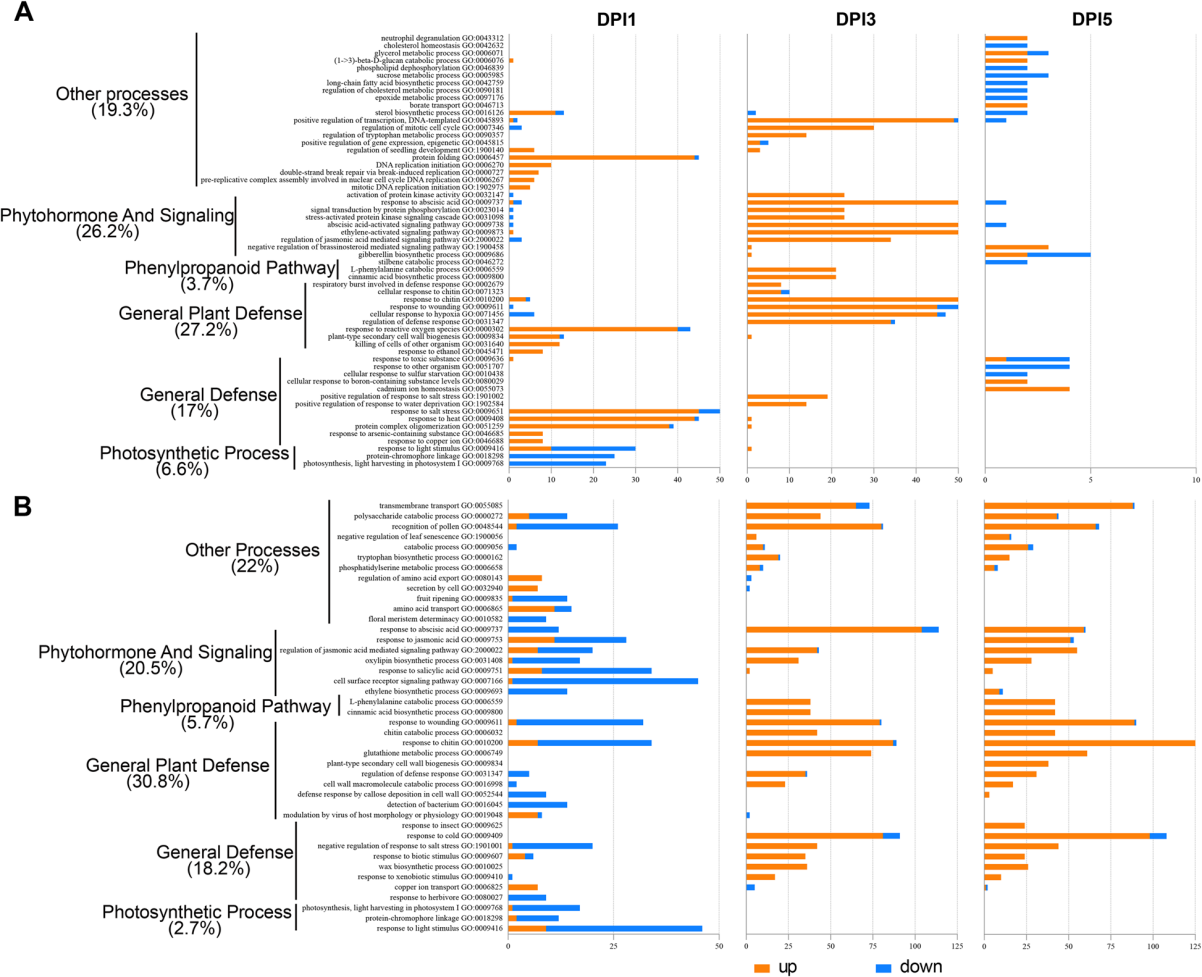
Significantly enriched gene ontology (GO) showing in inoculated vs control at different time points in Xinong 511 (**A**) and Aikang 58 (**B**) varieties. The y-axis represents significantly enriched GO processes which were enriched (FDR < 0.05) at least one time point of infection (1, 3 and 5 dpi). The x-axis indicates the total number of genes annotated to each GO process. Orange sections represent up-regulated genes while blue sections represent down-regulated genes

**Fig. 5:**
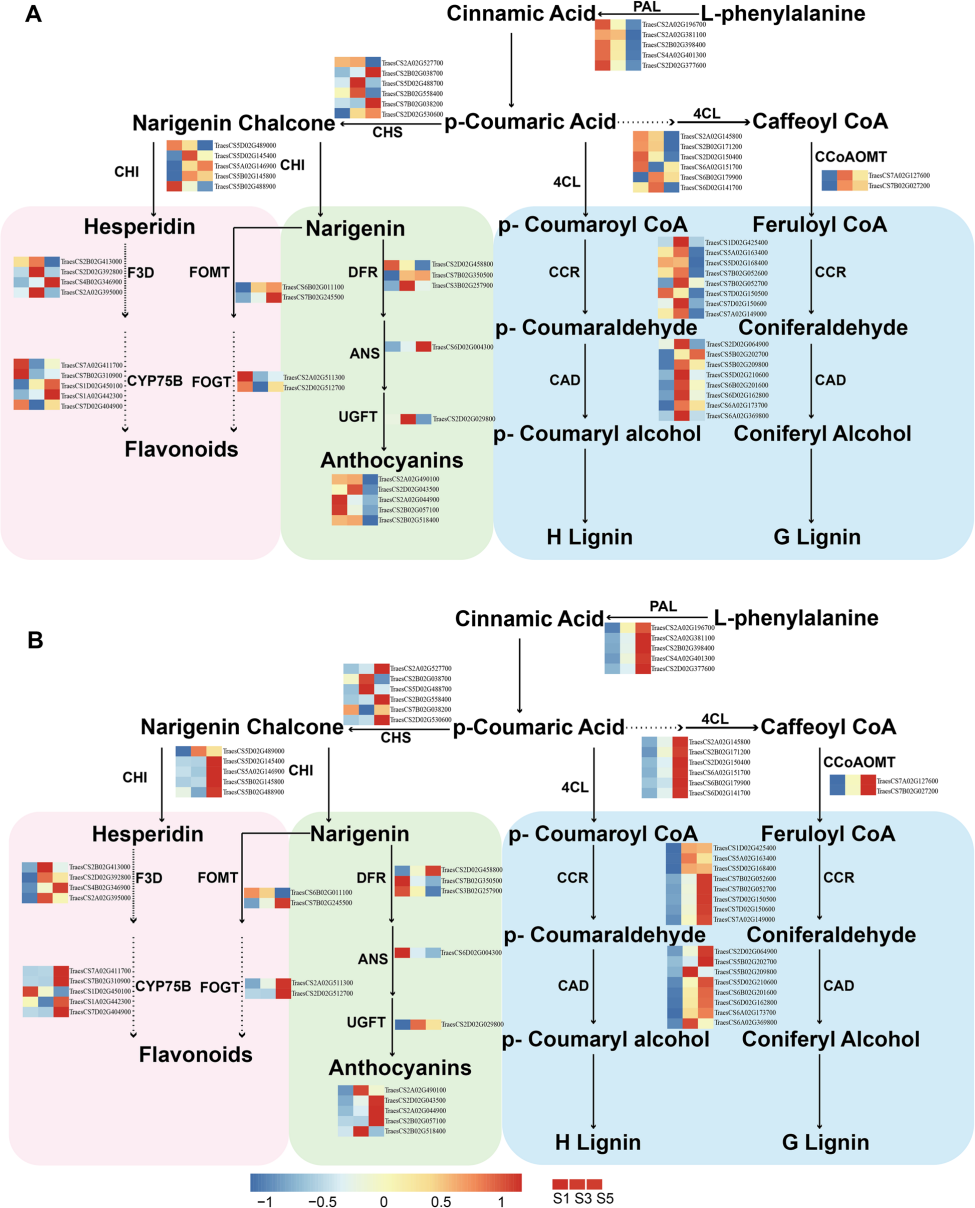
Phenylpropanoid pathway in Xinong 511 (**A**) and Aikang 58 (**B**) in resistance to *F. graminearum.* Enzymes are indicated in uppercase letters. PAL (phenylalanine ammonia-lyase); 4CL (4-coumarate: CoA ligase); CCoAOMT (caffeoyl-CoA O-methyltransferase); CCR (cinnamoyl-CoA reductase); CAD (cinnamoyl alcohol dehydrogenase); CHS (chalcone synthase); CHI (chalcone isomerase); CHR (chalcone reductase); FOMT (Flavonoid 4′-O-methyltransferase); ANS (anthocyanidin synthase also called LDOX, leucoanthocyanidin dioxygenase); ANS (anthocyanidin synthase); UFGT (UDP-flavonoid glucosyltransferase); AGT (Anthocyanidin-3-O-glucoside rhumnosyltransferase); DFR (Dihydroflavonol 4-reductase); F3D (Flavonoid 3-dioxygenase); FOGT (Flavonoid-O-glucosyltransferase); CYP75B (Flavonoid 3’-monooxygenase CYP75B). The color block represents the normalized value of FPKM. Pink, green and blue squares represent the synthesis of flavonoids, anthocyanins and lignans respectively. This figure was adapted using a previously described according to Ranjan and Ferrer (Ferrer et al. 2008; Ranjan et al. 2019)

During the flowering and ripening stage, XN511 and AK58 were selected for investigation during two winter wheat growing seasons (2020–2022). Cultivar XN511 consistently exhibited a percentage of symptomatic spikelets (PSS) of less than 20% over two consecutive years, was classified as an R line, whereas the AK58 showed a PSS of approximately 80%, was designated as an S line (Fig. 1B and C). To reveal the phenotypic differences and between the two types of materials post-inoculation with *F. graminearum*, the single floret spikelet inoculation method (SFI) was employed and infection progress was monitored over 14 days in *Fusarium*-inoculated spikelet and floret, respectively (Figs. 1A and 2). Consistent with the results reported previously (Fig. S1) (Brown et al. 2010), initial symptoms of FHB appeared on the palea and ovary at 1d post inoculation (dpi) as light brown lesions surrounding the inoculation site (Figs. 1A-1d, 2A-1d and B-1d). At 3 dpi, typical water-soaked lesions began to appear on the lemma at the inoculated floret on both two lines (Fig. 1A-3d). However, distinct differences were observed in the rachis: in S lines, fungal hyphae had broken through the rachis node, and extensive tissue colonization occurred along the rachilla and ovary (Fig. 2A-3d), whereas in the R lines, only small, localized brown spots were observed at the same point (Fig. 2B-3d). At 5 dpi, the differences between the two lines became more pronounced: the fungal hyphae had extended into the rachis internode in AK58 (Fig. 2A-5d), but in XN511, the hyphae were restricted to the ovary and rachis node of the inoculation site (Fig. 2B-5d).

Furthermore, paraffin sections analysis revealed no significant differences in the cellular structure of rachis, suggesting that the difference in pathogen spread within the rachis between the two lines are unlikely to be caused by the floral or rachis tissue structure (Fig. 1D). We thus propose that the rachis is a critical site for plant defense against FHB, and performed transcriptome sequencing on rachis tissues inoculated with either the pathogen and sterile distilled water (SDW) at three early time points (1 dpi, 3 dpi, and 5 dpi) showing distinct differences in resistance between the two lines. Over the following days, *F. graminearum* continued to spread in the rachis, and it was observed that in the susceptible material, the area outside the rachis infected by the mycelium had become brown and the rachis internodes were also filled with a large number of green mycelia (Fig. 2A). Compared to the susceptible material, fungal hyphae also appeared between the tassels in XN511, but over time the tassel at the inoculation site becomes necrotic, preventing hyphal expansion (Fig. 2B).

### The crucial role of varietal differences under *F. graminearum* infected

A total of 411.42 Gb of clean data from 36 samples of XN511 and AK58 against the ChineseSpring transcriptome showed that 1 359. 0 million reads (92.8%) were mapped in total and 1 128.3 million (83.0%) were mapped uniquely, and the value of Q20 (~ 97%) and Q30 (~ 94%) indicated that the quality of the sequencing data was sufficient to support further transcriptome analysis (Table S1). RNA-seq data were normalized to fragments per kilobase per million mapped reads (FPKM) values to quantify transcript expression, and to reduce the effect of transcriptional noise, genes with FPKM > 1.0 and expressed in two samples were used for subsequent analysis. To understand the transcriptional dynamics of the *F. graminearum* infection process of in resistant and susceptible materials, a hierarchical clustering and a principal component analysis (PCA) were performed on all samples (Fig. 3). The clustering tree diagram results showed that these samples could be initially divided into two categories according to the materials, and then, in each category, the different developmental stages were distinguished (Fig. 3A). Based on the PCA results (Fig. 3B), a high correlation between varieties was observed. Within the same variety, samples at the same developmental stage, both inoculated and control, also showed a high degree of correlation. This result is consistent with the results of the clustering dendrogram, suggesting a highly similarity in their transcriptional programs. At the early developmental T1 and C1 stage (designated S1, and T3 and C3 were designated S3, T5 and C5 were designated S5), the treatments (inoculated and control) of the two varieties exhibited lower correlations with stages S3 and S5, the latter two showing higher similarities in comparison. It was also observed that in R lines, the later stages (S3 and S5) of both inoculated and control treatments had higher correlations than in susceptible materials, indicating that susceptible materials underwent a strong induction after *F. graminearum* infection. In conclusion, hierarchical clustering and PCA analysis confirmed that the greatest variation occurred from S1 to S3 phases, and that differences between varieties were the main determinant of the variance in disease resistance observed between the two wheat varieties.

### XN511 and AK58 transcriptomes upon *F. graminearum* infection reveal unique gene expression

Comparative analysis of DEGs was performed between the two cultivars and within the same variety between *Fusarium*-infected and mock-inoculated samples, and the up- and down-regulated genes of each group are shown in Fig. S2. In compared with the control groups, the R line exhibited the highest number of DEGs at stage S3 and the lowest at stage S5 (Fig. S2A), indicating that XN511 had returned to a steady state in response to fungal infection. In contrast, the S line demonstrated a pronounced transcriptional response with the greatest number of DEGs at stage S5 with 5998 and the least at stage S1 with 2493 DEGs. These results show that *Fusarium-*infection induces significant changes in wheat gene expression, which are particularly pronounced in the susceptible cultivar. A venn analysis of the DEGs was performed and shows that the phenomenon was consistent with the previous findings (Fig. S2B). The observed differences in expression profiles reflect distinct responses of the R and S lines to *F. graminearum* infection.

To further determine the function of DEGs and to distinguish differences between groups, we focused on comparisons between inoculated and control samples to look for biological processes associated with disease resistance. The wheat gene IDs identified by the DEGs were used to perform gene ontology (GO) enrichment analysis, and the top 20 biological processes terms in each group are shown in Fig. 4A and B, and a false discovery rate (FDR) value of 0.05 was used to identify significantly regulated GO terms (Table S2). In the resistant variety XN511, upregulated genes predominantly concentrated in GO terms associated with immunity, phytohormones, and plant defense-related processes (Fig. 4A). The DEGs identified at the 1 dpi stage indicated an early response to *F. graminearum* infection, and the GO terms for this stage were focused on related pathways such as the general plant defense and other general defenses, whereas at later stages, there is enrichment of GO terms associated with more general defense and immunity processes, such as “stress-activated protein kinase signaling cascade”, “response to chitin” and “regulation of jasmonic acid mediated signaling pathway”, revealing a comprehensive response to *F. graminearum* infection. Conversely, at the 5 dpi stage, both the quantity of DEGs and the outcomes of GO analyses collectively suggested that the response to *F. graminearum* had reached its zenith and was subsequently entering a phase of decline. Down-regulated genes are enriched mainly for terms related to photosynthesis and chloroplast biology, a phenomenon that is often justified because cell death is accompanied by a reduction in photosystem and photosynthetic efficiency, which is also part of the defense and yield trade-off to gain resources for the immune response and slow down sugar and nutrient production because they might serve as a source for pathogen survival and multiplication (Dangl and Jones 2001). Strikingly, the GO expression patterns in the susceptible variety AK58 exhibited marked differences when compared to XN511 (Fig. 4B): in the early stage (1 dpi), GO terms related to plant defense, phytohormones, and signaling, such as “cell surface receptor signaling pathway”, “regulation of jasmonic acid mediated signaling pathway”, “response to salicylic acid” and “detection of bacterium” underwent significant changes, but predominantly characterized by gene downregulation. Similar to XN511, genes associated with photosynthesis also showed a trend of downregulation during this period. At the 3 dpi stage, GO terms were primarily elevated in relation to plant defense, signaling, and the phenylpropanoid pathway, predominantly exhibiting gene upregulation, indicating that AK58 had commenced its response to *F. graminearum* infection. The GO terms at the 5 dpi stage was similar to those at 3 dpi, but with a higher number of DEGs, demonstrating a sustained response of AK58 to *F. graminearum* infection from 3 to 5 dpi.

### DEGs involved in phenylpropanoid pathway was significantly up-regulated expressed in susceptible cultivar compared to resistant cultivar

The phenylpropanoid pathway plays an integral role in plant defense, predominantly by generating a multitude of secondary metabolites to assist plants in coping with various environmental stresses (Ramaroson et al. 2022). To comprehensively understand the effect of FHB on the expression of genes involved in the phenylpropanoid metabolic pathway in wheat, we constructed a network model of the wheat phenylpropanoid pathway (Fig. 5). In this study, we observed differential regulation of transcripts related to the phenylpropanoid pathway in resistant (Fig. 5A) and susceptible (Fig. 5B) wheat varieties. During the S1 stage of disease resistant materials, we observed an initial high expression of the PAL and 4 CL (4-coumarate: CoA ligase) genes, which subsequently decreased. Throughout the entire disease resistance process, there were no significant differences between the treated and control groups. However, in the disease-susceptible materials, the expression of these genes was found to increase following *Fusarium*-infected, with a marked difference between the control and treated groups. We speculate that the rapid response and display of disease-resistant phenotype in the resistant materials at the early stage of pathogen invasion might be closely associated with the high expression of PAL and 4CL genes in the initial phase of disease resistance. Lignin, a crucial component of the cell wall, confers mechanical strength and impedes pathogen entry (Lee et al. 2019). We noted a significant up-regulation in the expression of CCR (cinnamoyl-Co A reductase) and CAD (cinnamoyl alcohol dehydrogenase) during pathogen infection, with a more rapid response in the cultivar XN511. Variations in the expression of CCR and CAD genes between the two varieties might lead to differences in lignin content, thereby impacting the plant’s disease resistance response. The synthesis of anthocyanins and flavonoids, vital branches in the phenylpropanoid metabolic process, also plays significant roles in plant defense. Anthocyanins aid plants in warding off pathogens such as fungi, bacteria, and viruses (Kaur et al. 2023), while flavonoids can directly inhibit pathogen growth or disrupt their interactions with plants (Shaw et al. 2006). The differential expression of genes related to anthocyanin and flavonoid synthesis pathways between the varieties might also be a contributing factor to the variations in the resistance phenotype post-*F. graminearum* infection. The expression of the AK58 line genes gradually increases, but the XN511, with its rapid responses to *F. graminearum* infection, exhibits higher expression levels in the initial stages of infection, gradually decreasing by S5 phase, marking the end of the resistance responses. The expression of anthocyanin-related genes was also observed, with five anthocyanin-related genes showing higher expression in the early stages of infection in the R line, whilst the expression of related genes in the S line increased progressively with the severity of pathogen attack. Overall, *Fusarium-*infected plants up-regulated the phenylpropanoid metabolic pathway, with XN511 demonstrating a notably swift response, thus rapidly initiating a defense reaction, possibly underpinning its resistance to the pathogen.

### Weighted gene co-expression network analysis of DEGs in resistant cultivar

**Fig. 6:**
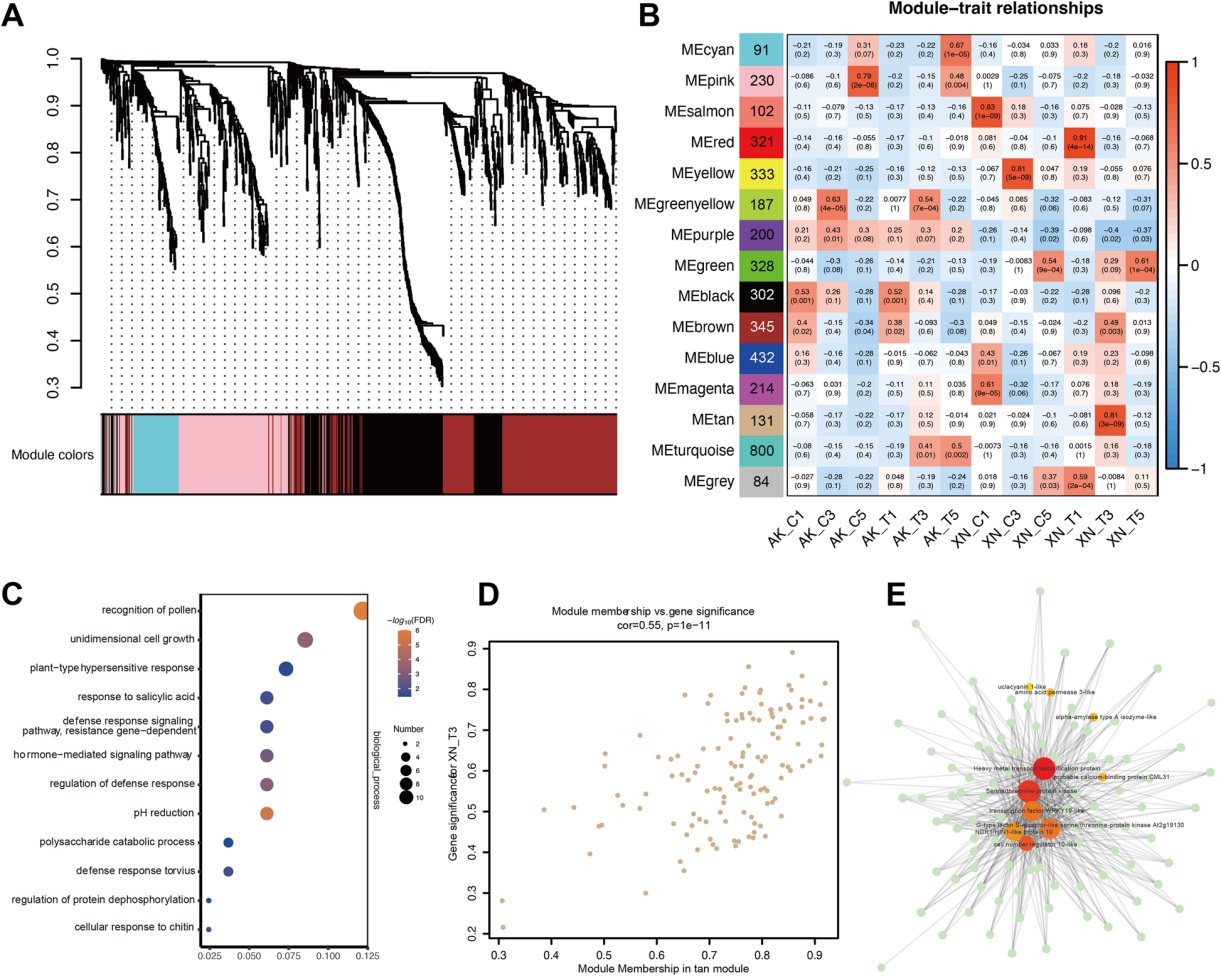
Weighted gene co-expression network analysis (WGCNA) of differentially expressed genes (DEGs) and module MEtan. **A** Hierarchical clustering tree based on co-expression network, each leaf in the tree represents on gene. All genes outside any suitable modules were clustered into grey module; **B** Correlation analysis between the sample and each module. The numerical *Pearson*’s correlation coefficient and corresponding *P*-value in parentheses were marked in each module. The color scheme, from red through white to blue, shows the levels of correlation, from high to low. Each row corresponds to a module. The name of modules is indicated on the left. Each column corresponds to a specific sample. **C** The GO analysis in module tan. **D** Correlation between gene significance and module membership of tan module. **E** The correlation network of module of tan. A gene network is constructed by WGCNA, in which each node represents a gene, and the connecting line (edge) between genes represents the co-expression correction. The size and color of each circle represent the number of edges

To further investigate the key determinants of disease resistance in the R line, the researchers performed a WGCNA on the DEGs in XN511. A total of 4100 genes were analyzed through WGCNA, resulting in the identification of 16 distinct modules, each marked by a different color (Fig. 6A, B, and Table S4). The MEturquoise module associated with the S-treated group (AK_T) clustered the most genes (800, ~ 20%), which is in consistent with early DEGs (Fig. S2A). This indicates a robust response of AK58 to *F. graminearum* infection. Almost 28% (1155) of genes were clustered at development stage-specific modules (MEcyan, MEpink, MEgreenyellow, MEblack, MEbrown) in S line, but only one module (MEgreen, 328 genes) was associated with XN511 development. A total of 200 genes clustered in the MEpurple module of varietal differences, the variety-specific expression patterns of these genes probably reflected the key functions they play. This was consistent with early hierarchical clustering (Fig. 3A) and PCA (Fig. 3B), and gene expression in the species was highly conserved, followed by the differences between the developmental stages. Based on the results of GO and DEGs analyses, the disease resistance response in XN511 was predominantly observed in stages S1 and S3. Consequently, our attention was drawn to two specific modules: MEtan (tan) and MEred (red) during this investigation. Analysis of the relationships between module and sample significance revealed a strong positive correlation between XN_T1 and the MEred module (*r* = 0.91), and between XN_T3 and the MEtan module (*r* = 0.81) (Fig. 6B). GO enrichment indicated that genes in the red module are involved in various biological processes, with relevant GO terms including responses to heat, reactive oxygen species, salt stress, and ethanol (Fig. S3B). These genes are likely crucial in the early stages of plant pathogenic infection, activating defense mechanisms and signal transduction to ensure the proper progression of local or comprehensive plant defense responses. Conversely, genes in the tan module are mainly associated with plant disease resistance-related GO terms, such as plant hypersensitive response, regulation of defense response, response to salicylic acid, defense against viruses, and defense response signaling pathways (Fig. 6C). This demonstrates that genes in this module play a vital role in initiating local or comprehensive defense and establishing systemic acquired resistance, essential in plant defense responses. Further, we calculated the correlation values between module membership and gene significance (Fig. 6D and S3A), and used CytoHubba to select the top 10 hub genes, constructing gene regulatory networks in the red and tan modules using Cytoscape (Fig. 6D and Fig. S2C). We discovered that the top 10 hub genes in the MEred module predominantly encode heat shock proteins of approximately 20 kDa (Fig. S3C). In contrast, the MEtan module features genes associated with heavy metal transport/detoxification proteins, serine/threonine receptor kinases, WRKY transcription factors, and proteins linked to calcium signaling. Notably, among these, the detoxification proteins have been demonstrated to confer resistance to *F. graminearum* in Arabidopsis (Wang et al. 2021).

### The role of CYP and HMA proteins in plant resistance to *Fusarium* head blight

**Fig. 7:**
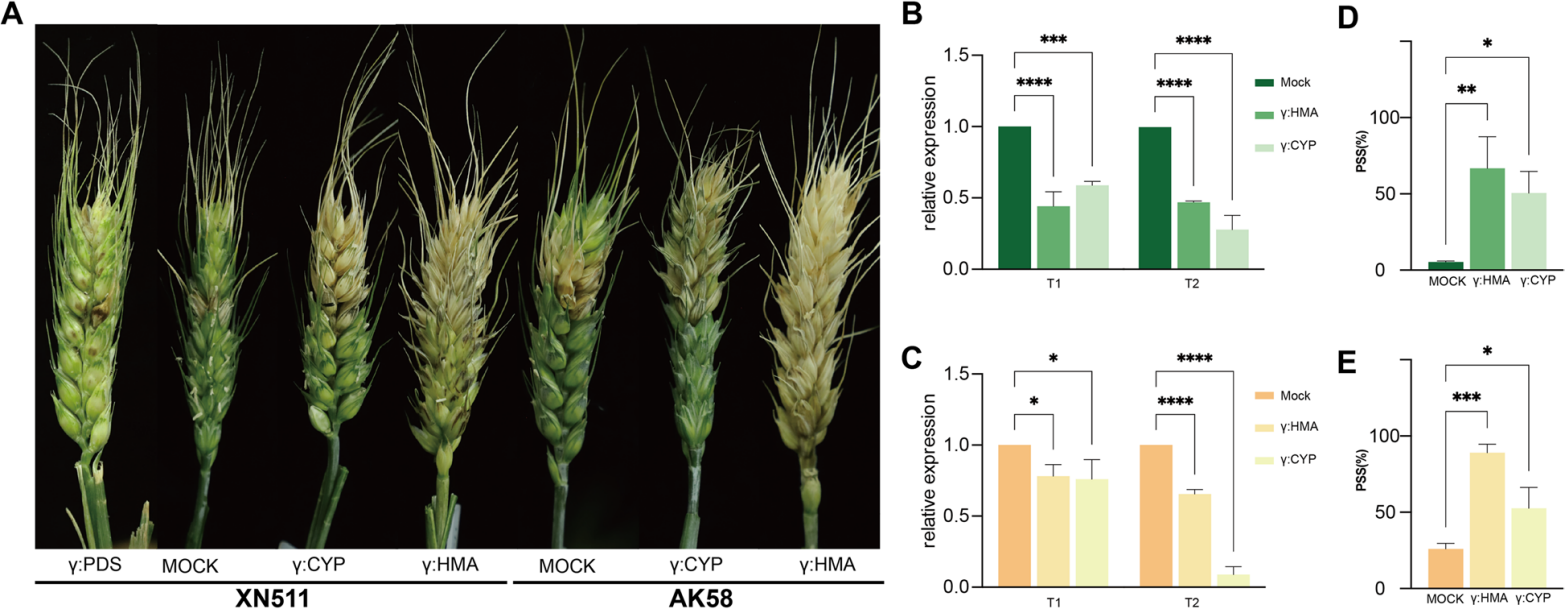
The silence of *TaCYP* and *TaHMA* reduces the resistance of plants to *Fusarium* head blight (FHB). **A** Flag and inverted second leaf were inoculated with BSMV-*TaPDS,* resulting in pronounced photobleaching of spikelets; after BSMV-γ inoculation, disease phenotypes with typical water-soaked lesions appeared on the lemma on the 3 th days after inoculation with *F. graminearum*. Photographs were taken ten days after inoculation with *F. graminearum*. The expression patterns of genes *TaCYP* and *TaHMA* in spikelet samples from the Xinong 511 (**B**) and Aikang 58 (**C**) varieties at both 7 dpi (T1) and 10 dpi (T2), following inoculation with *F. graminearum.* The Percentage of symptomatic spikelets (PSS) in mock and silenced plants after *Fusarium*-inoculated with XN511 (**D**) and AK58 (**E**). Genes expression levels were assessed by qPCR and data were normalized to the wheat housekeeping gene glyceraldehyde-3-phosphate dehydrogenase (*GAPDH*) (Beccari et al. 2011) expression level. Data represent the mean ± SD (*n* = 3 independent replicates). Significant differences are indicated with asterisks. Data are presented as means ± SD (*P < 0.05, **P < 0.01; ***P < 0.001; ****P < 0.0001, Student’s t-test)

To confirm that the phenylpropanoid pathway and the hub genes identified by WGCNA analysis were present in *Fusarium*-infected wheat, we selected phenylpropanoid pathway-related and heavy metal-associated domain genes, *TraesCS2B02G006200* (*TaCYP*) and *TraesCS5B02G030300* (*TaHMA*). We constructed silencing vectors for these genes using viral-induced gene silencing (VIGS) with barley stripe mosaic virus (BSMV) in order to reduce their expression levels in wheat ears, which was later verified by gibberellic acid inoculation of the silenced strains. On day 7 post-inoculation, distinct phenotypic differences became evident (Fig. 7A). In XN511, silenced *TaPDS* plants, as well as controls, showed few symptomatic florets, whereas silenced plants of *TaCYP* and *TaHMA* significantly increased on PSS (Fig. 7D). RT-qPCR analysis revealed substantial suppression of *TaCYP* and *TaHMA* in XN511, with silencing efficiencies of 38–82% and 45–70%, respectively (Fig. 7B). In susceptible plants, AK58 had significantly fewer diseased florets compared to silenced *TaCYP* and *TaHMA* plants (Fig. 7E), and RT-qPCR analysis showed that both genes were also reduced in AK58, with silencing efficiencies ranging from 14–90% for *TaCYP* and 12–36% for *TaHMA* (Fig. 7C). Additionally, silencing of *TaHMA* appeared to have a greater effect on plants, as evidenced by a higher PSS in the spikes of the silenced plants. Taken together, silencing of the *TaCYP* and *TaHMA* can significantly reduce immunity to *F. graminearum* in wheat, increasing susceptibility in resistant plants and exacerbating disease severity in susceptible ones.

### Population transcriptomics reveals the role of phenylpropanoid pathway genes in plant disease resistance

**Fig. 8:**
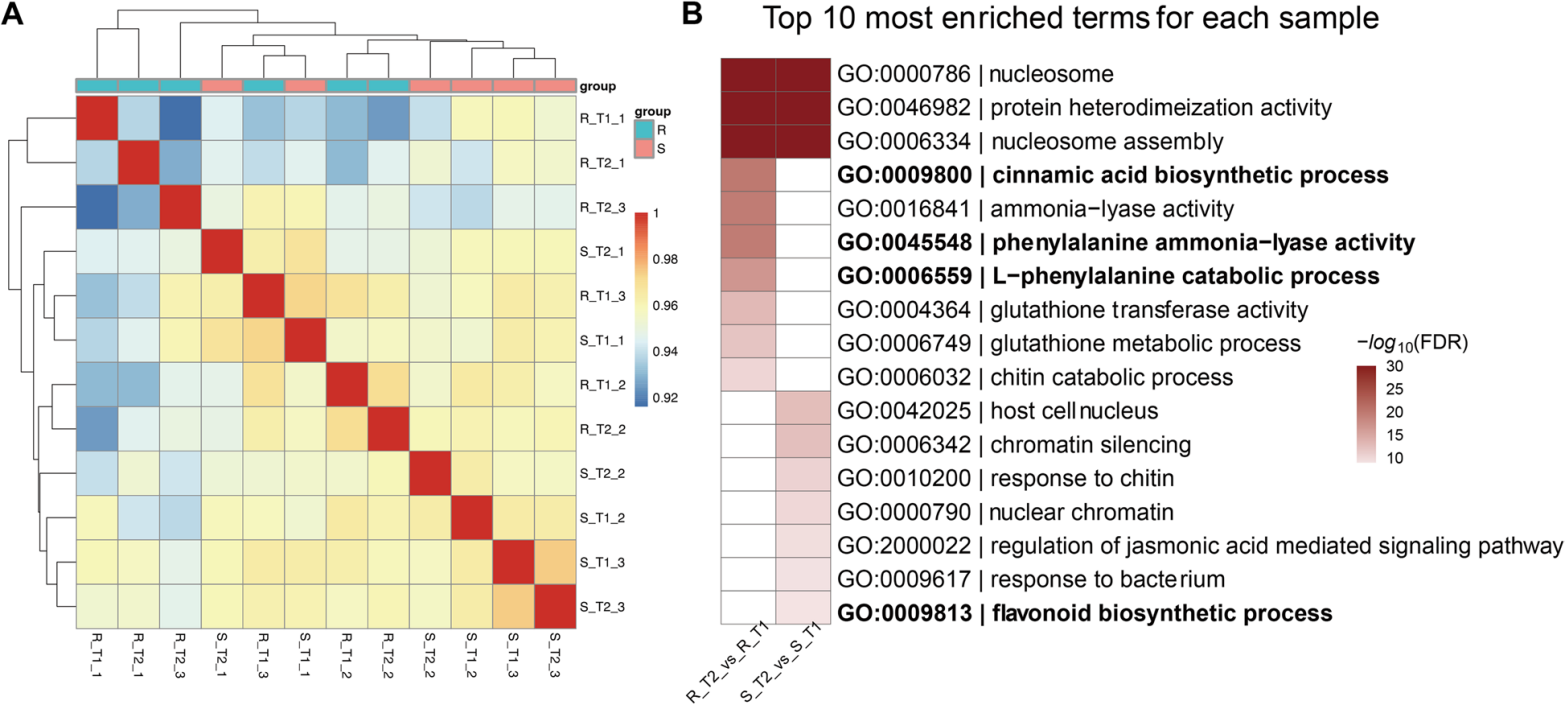
The sample correlation heatmap and GO analysis (**A**). The sample correlation heatmap (**B**). GO analysis plot between different treatment groups

To confirm that the results of the previous study were not due to differences in the genetic background of the two cultivars, we performed paired-end RNA-seq on disease-resistant and susceptible lines in the RILs constructed from a cross between XN511 and AK58, using three biological replicates for each sample (Table S6). The sample correlation heatmap analysis (Fig. 8A) displayed that the correlation between the transcriptomes was greater than 90%, indicating they share the same genetic background. PCA of the samples also displayed the similar clustering (Fig. S4A), with most samples being distinguished based on their resistant and susceptible lines. The results showed that the number of expressed genes was higher in susceptible samples compared to resistant samples (Table S7), and that in AK58, the number of expressed genes in the treatment group was higher than that in the control group. This suggests that, upon *F. graminearum* infection, the susceptible wheat materials experience a stronger induction compared to the resistant materials.

DEGs analysis between susceptible and resistant wheat lines at the same time as well as between different time points within the same wheat line (Table S8 and Fig. S4B). The identified DEGs were analyzed for GO analysis (Table S9). The results indicate that the GO terms in the DEGs between resistant and susceptible lines at different times are presumably similar. The DEGs at both time points showed intersections in metabolic and enzyme activity, nutrient and substance transport, as well as storage and synthesis. R_T1vsS_T1 is particularly concerned with mitochondrial function, cell death, and specific environmental response processes; whereas, R_T2vsS_T2 encompasses processes that are more related to growth and development, as well as cell signaling pathways. Similarly, DEGs between susceptible and resistant wheat lines at the same time points were analyzed (Fig. 8B), and the results showed that both time points had intersecting functions related to nucleosome activity. However, in R_T2vsR_T1, more genes were involved in processes related to the phenylpropanoid pathway, whereas in S_T2vsS_T1, the genes were more concentrated in processes related to general plant defense mechanisms, such as response to chitin and hormone regulation. DEGs analysis between the two parental lines under mock-inoculated conditions also supported the above findings (Table S10).

## Discussion

FHB has caused widespread yield losses and quality degradation in crops, while mycotoxins produced during *F. graminearum* infection contaminate crops, posing a serious threat to the safety of humans and domestic animals (Perincherry et al. 2019). Building upon Brown (Brown et al. 2010) description of the colonization process of *F. graminearum* in susceptible plants, this study further investigates the interaction between plants and pathogens. We traced the colonization of *F. graminearum* in both susceptible and resistant wheat spikelets and rachis and found that the infection by the pathogen began in the anthers before invading the wheat spike rachis (Fig. 1A). However, significant differences were observed in the colonization patterns within the rachis (Fig. 2). The fungus could spread easily and extensively from the inoculated spikelet into the rachis and adjacent spikelets in AK58, but was effectively blocked from spreading in XN511. Fluorescence scanning microscopic examination revealed that fungal growth was inhibited within the inoculated site on the spike rachis in XN511, and the spread of the fungus was blocked by the internodal tissues, and the fungal growth was prevented from spreading widely in the rachis and adjacent spikelets. These results highlight the critical role of the spike rachis in wheat responses to pathogen infection, emphasizing the complexity of plant-pathogen interactions and the important role of the rachis in defensive response.

The previous findings revealed that rapid establishment of transcriptional reprogramming is critical for robust resistance (Mine et al. 2018). Consistent with this, our study also revealed significant differences in the response of resistant and susceptible materials to *F. graminearum* infection. The resistant material rapidly completed its response to pathogen invasion and mounted a disease resistance reaction within a short timeframe (1 dpi-3 dpi), with the dpi1 stage primarily focused on responding to pathogen invasion and 3 dpi mainly involving disease resistance reactions. In contrast, the susceptible material failed to respond promptly to the pathogen invasion, leading to a comprehensive infection process by the pathogen. Two important questions are, is early-stage transcriptional reprogramming important in plant defense? What leads to significant differences between resistant and susceptible material in response to *F. graminearum* infestation? In plants with the common genetic components, early stages of recognition and transcriptional reprogramming lead to the establishment of comprehensive defense mechanisms and the expression of resistance to disease (Mine et al. 2018; Ranjan et al. 2019). Our results also indicated that the resistant material undergoes early establishment of transcriptional reprogramming, specifically in response to plant defense-related gene ontologies, while the susceptible varieties do not exhibit such early responses (Fig. 4). In addition to this, differences in the colonization and spread of pathogenic fungi in the spike rachis of different resistant wheat varieties may contribute to variability in early pathogen recognition and response (Fig. 2). Although the precise factors leading to these differences in fungal colonization between resistant and susceptible varieties remain unclear, host susceptibility factors are key determinants in pathogen growth and disease progression within plant tissues, as suggested by Kazan and Gardiner (2018) and studies on polyamine genes in susceptible plants (Chetouhi et al. 2016). Further research into the behavior of pathogens during infection will be vital.

Pathogens such as fungi, bacteria, nematodes and insects intercept the photosynthate produced by plants. Plants, in turn, have evolved sophisticated mechanisms to perceive such complex mechanisms, and to translate that perception into an adaptive response (Dangl and Jones 2001). For instance, plants generate a complex network of infection by producing defense-related hormones, pathogenesis-related proteins, reactive oxygen and proteins involved in cellular detoxification (Kazan et al. 2012). This intricate network demonstrates the sophistication of plant defense systems against a various of biotic threats. Identifying the key components of plant defense responses is essential for developing disease-resistant crop varieties. In this study we conducted into the wheat resistance mechanisms against *F. graminearum* by comparing opposing outcomes in two wheat lines in response to FHB. Our findings are consistent with these key points: (i), inter-varietal differences are the primary cause of phenotypic variations; (ii), plant defense responses are closely associated with high-amplitude transcriptional reprogramming within the early time window; (iii), reduced photosynthesis accompanies early plant responses to pathogens; (iv), pathogen attack leads to significant changes in wheat gene expression, with susceptible varieties showing more dramatic changes; (v), we observed transcriptional reprogramming of the phenylpropanoid pathway during *F. graminearum* infection; and (vi), detoxification proteins play a crucial role in plant defense responses. Overall, the transcriptome studies allowed us to uncover a novel resistance mechanism of wheat to *F. graminearum* Despite similar infection patterns across wheat spike, significant differences in gene expression within the spike rachis highlight the uniqueness of each variety’s resistance mechanisms.

The phenylpropanoid pathway is one of the most extensively studied in plant secondary metabolism (Dixon et al. 2002; Ramaroson et al. 2022), and a wide range of secondary metabolites like flavonoids, tannins, lignin, and other phenolic compounds, are crucial for plant defense against pathogen invasion (Perincherry et al. 2019). The accumulation of these phenolic compounds is typically correlated with plant tolerance and is widely distributed in numerous metabolic and physiological processes in plants (Kumar et al. 2019; Sharma et al. 2019), and their biosynthesis is regulated by changes in the activities of key enzymes in the phenolic biosynthetic pathway, such as PAL, CHS, CHI. Importantly, the enhancement in the activity of these enzymes is often paralleled by an increase in the transcription of genes encoding these crucial biosynthetic enzymes (Chen et al. 2019; Zhou et al. 2018). Our research has established the crucial role of the phenylpropanoid pathway in plant defense. Plant disease resistance is associated with the early accumulation of several key enzymes in the phenylpropane pathway and verifies the function of cytochrome P450 involved in the metabolism of flavonoids in wheat FHB. CYP protects plants from harsh environmental conditions by enhancing the antioxidative activity of compounds like flavonoids (Rao et al. 2020), and, as oxidoreductases, catalyze NADPH-/O2-dependent hydroxylation reactions in primary and secondary metabolism and play a central role in the detoxification of exogenous substances (Kazan and Gardiner 2018). Additionally, other metabolites in the phenylpropanoid pathway contribute significantly to phytohormone regulation, signaling, strengthening cell wall integrity, and inducing systemic acquired resistance (Sharma et al. 2019). Collectively, these mechanisms enhance plant resistance against a range of biotic and abiotic stresses.

When plants are infested with pathogens, the mineral element balance in the body is altered, potentially affecting the activity of metal cation-dependent enzymes and thus altering plant metabolic pathways (Calla et al. 2014; Cesco et al. 2020). The *NRAMP* family in plants is responsible for the uptake, translocation, and detoxification of transition metals (Yang et al. 2022a, b). This phenomenon underscores the potential role of mineral elements in resistance to pathogens. For example, the heavy-metal transporter *OsNRAMP1* in rice modulates the immune response of plants by regulating reactive oxygen species (ROS) homoeostasis (Chu et al. 2022). The heavy-metal transporter also plays key roles in plant defense, with proteins like *OsNRAMP2* and *OsNRAMP6* involved in the transport of iron and manganese, contributing positively to pathogen defense (Li et al. 2021; Peris-Peris et al. 2017). Our study further highlights the important role of heavy-metal transporter of the hub gene of the MEtan module in WGCNA in plant defense against pathogen, offering new insights for plant pathology and disease-resistant crop breeding.

According to previous mapping results (Yang et al. 2024), *QFhb.nwafu-5B* was located between SNP5B-4 and SNP5B-6, whereas *QFhb.nwafu-7A* was situated between SNP7A-4 and SNP7A-25. The gene *TraesCS5B02G030300 (TaHMA)* is located at position 33,555,852–33,557,509 on chromosome 5B in the CS genome, which is clearly outside the physical interval of *QFhb.nwafu-5B*. This result is reasonable, as disease resistance in plants is a complex trait that is typically governed by networks of multiple gene s rather than by a few individual genes. As noted previously, the genetic populations used in earlier studies were limited by both a low number of generations and a relatively small population size, making them unsuitable for precise genetic mapping. In addition, the previous results were based on genetic distance, and whether the genetic distance is consistent with the physical distance still needs to be further verified by expanding the genetic population size and increasing the number of generations. Transcriptome analysis screens DEGs to find genes and pathways related to disease resistance. Although *TaHMA* is not located within the *QFhb.nwafu-5B* QTL interval, it may still play a functional role in the FHB resistance pathway and contribute to the defense response. Our results indicate that the wheat rachis is a key component for resistance to FHB, and this study provides new insights into wheat and *F. graminearum* interactions, but the mechanisms require further investigation. Meanwhile, plant disease resistance is associated with transcriptional reprogramming in response to early pathogen infestation. In addition, preliminary validation of the *TaCYP,* which is involved in the flavonoid biosynthesis within the phenylpropanoid pathway, and *TaHMA*, the heavy metal-associated domain protein**,** genes showed that they inhibit FHB, and their variety improvement and breeding can be further exploited through studies such as gene editing or transgenesis. Therefore, based on previous studies as well as a novel perspective, this study comprehensively evaluates the infestation pattern and defense characteristics of *F. graminearum* in wheat, while also identifying directions for future research.

## Conclusion

This study indicates that the rachis is a critical site for XN511 defense against FHB. Compared with AK58, XN511 exhibits an earlier response to FHB infection. Transcriptome analysis revealed that early transcriptional reprogramming and the phenylpropanoid metabolic pathway play an important role in the disease resistance mechanism, and that transient silencing of *TaCYP* and *TaHMA* genes significantly reduces wheat resistance to FHB. This research not only enriches the genetic foundation of resistance to wheat FHB but also provides valuable resource for disease-resistant breeding programs.

## Materials and methods

### Plant material and pathogen inoculation

The resistant wheat variety XN511 and a susceptible variety AK58 were planted at Yangling, Shaanxi Province, China (34°16ʹ N, 108°4ʹ E) during the 2020—2022 winter wheat growing season, and managed according to general field management. Seedlings and adult plants of the RNA-induced gene silencing experiments were grown in an incubator and greenhouse, respectively. The *F. graminearum* strain “PH-1 (NRRL 31084)” and was kindly provided by College of Plant Protection (Northwest A&F University, Yangling, China). In this research, a *F. graminearum* strain PH1 labelled with GFP was used to accurately observe the response of different resistant materials to at different inoculation periods. This GFP-labelled *F. graminearum* was comparable to wild-type *F. graminearum* in terms of infestation efficacy. Cultivation of *F. graminearum* strains and plant inoculation were performed as described in Yang (Yang et al. 2022a). Spores were quantified and suspensions adjusted to a final concentration of 5 × 10^5^ spores/mL in SDW by blood counting chamber. Plant heads of 6-week-old wheat plants were inoculated at the fourth spikelet from the top with 10 μl of conidium suspensions or SDW.

### Infection assays with flowering wheat heads and morphological observation

Inoculated wheat heads were examined for diseased spikelets at 18 or 21 dpi to estimate the disease index, defined as the PSS. The mean of the disease index was estimated with data from three independent replicates with at least 10 randomly chosen wheat heads examined in each replicate. We inoculated wheat spikes using the SFI and sampled at 2- to 3-day intervals (Yang et al. 2022a, b). The samples were subsequently photographed using a Zeiss Discovery.V20 Stereo Microscope Stereoscope, ensuring that each set of samples contained at least five inoculated wheat spikes.

Paraffin sections analysis was performed as described previously (Liu et al. 2021). Briefly, Spike rachis node samples from the XN511 and AK58 lines (not inoculated with the pathogen) were collected and fixed in fixed in formalin-acetic acid-alcohol solution (FAA: a solution of formaldehyde, acetic acid, and ethanol) for 24–48 h. After undergoing a graded ethanol dehydration series and being destained in a series of xylene solutions, the tissues were embedded in paraffin. The embedded rachis internode tissues were trimmed and sectioned using a microtome into 10-mm-thick slices. The sections were sequentially stained with safranin O and fast green staining solution (G1031, https://www.servicebio.cn). The stained slides were cleared with xylene, mounted using neutral gum, and then observed under an Olympus BX-43 microscope (Japan).

### Sample collection and RNA sequencing

To reveal this variation, global transcriptome analysis was performed on spike rachis tissues collected from R and S lines at 1, 3, and 5 days after inoculation or mock inoculation. RNA-seq data were generated from 36 rachis samples of two varieties, consisting of three independent biological replicates for each treatment and mock. All samples were named using the abbreviations of the variety names and treatment, i.e. the abbreviations of XN511 and AK58 were XN and AK, respectively, and the abbreviations of noculated and control were T and C. Wheat rachis samples were collected from two varieties and two treatments at three time points: treated samples at 1, 3 and 5 dpi and control samples at 1, 3 and 5 dpi. Inoculation site wheat rachis was collected from, with 3–5 inoculated wheat heads were pooled together for each of the three biological replicates for RNA extraction. RILs material for population transcriptomics consisted of *Fusarium*-infected heads (4–5 per plant), and inoculated florets were collected at 24 h and 48 h, and marked accordingly. Post-inoculation, plants were assessed: those with less than 25% PSS were labeled as R (resistant), and those with more than 75% PSS were marked as S lines. 10–20 inoculated florets from resistant plants were pooled into R bulks, and those from susceptible plants into S bulks. These samples were immediately placed into liquid nitrogen and stored at −80 °C for RT-qPCR and transcriptome sequencing. RAN-seq libraries were sequenced to generate 150-nucleotide paired-end reads on an Illumina NovaSeq 6000 platform (Biomarker Technologies, Beijing). After removing the adaptors and low-quality reads with Trimmomatic tool (v0.39) (Bolger et al. 2014), the Hisat2 (v2.1.0) (Kim et al. 2019) was used to build index and align the paired-end clean data to reference genome. The wheat genome sequence data were acquired from the ChineseSpring v1.1 (http://plants.ensembl.org/Triticum_aestivum/Info/Index). Then, the Samtools (v1.3) (Li et al. 2009) was used to sort and convert the mapping results. The Featurecounts (Liao et al. 2014) was used to count the number of reads mapped to the reference genome and the FPKM value was used to quantitatively estimate the value of gene expression. DEGs were performed in the R environment (https://www.r-project.org/) with a package DESeq2 (Love et al. 2014), and the genes with log_2_FC (fold-change) > 1 or < − 1 and *p*-value < 0.05 were selected for follow-up analysis. The hierarchical clustering and PCA were performed by R packages Stats (v4.2.1) and Factoextra (v1.0.7), respectively. Using the *Triticeae*-Gene Tribe platfrom (http://wheat.cau.edu.cn/TGT/) (Chen et al. 2020) GO term enrichment tool, DEGs comparing treatment to control were used to identify enriched GO terms.

### Regulatory network construction by WGCNA

Co-expression networks were performed to screen gene modules or core genes linked to phenotypic traits of interest or target samples, using WGCNA (v1.7.1) (Langfelder and Horvath 2008) package in R. A total of 4100 genes were used for WGCNA analysis. The eigenvalues of each module were calculated and used to test the association with each variety, stage and treatment. Cytoscape (v3.10.1) (Shannon et al. 2003) was used for the visualization of the gene networks and the plugin CytoHubba (v0.1) (Chin et al. 2014) was used to filter the hub genes.

### Constructing the barley stripe mosaic virus vector and RNA-induced gene silencing

Using the cDNA of inoculated XN as template, the target sequence of the *TaCYP* and *TaHMA* genes were amplified by PrimerSTAR Max (R045B, Takara). The amplified product was ligated into BSMV vector BSMV-γ at the enzyme sites of *SpeI* and *Bam*HI vector and sequenced. Subsequently, the plasmid BSMV-α, BSMV-β, the recombinant plasmid BSMV-*TaCYP*, BSMV-*TaHMA* and positive control plasmid BSMV-*TaPDS* (photobleaching phenotype) were constructed into *Afrobacterium tumefaciens* stain GV3101 according to a previously described method (Sparkes et al. 2006). *N. benthamiana* and wheat infestation as described previously (Li et al. 2021; Zhang et al. 2022). In particular, the virus was first inoculated into four-week-old *N. benthamiana* leaves, and when the leaves exhibit chlorosis symptoms (typically within 3 to 7 days), it could be ground into juice friction inoculation on the leaves of wheat seedlings (in wheat three-leaf stage). Ten days post-inoculation, observation of photobleaching phenomenon on the fourth or fifth leaf of wheat seedlings inoculated with BSMV-*TaPDS* indicates successful virus emission, signifying readiness for subsequent inoculation steps. Next, the fourth leaves of the BSMV-*TaPDS* plants and the rest of the silenced plants were collected, ground again and inoculated onto the flag leaf and the penultimate leaf of wheat at the booting stage, and the photobleaching phenomenon should be observed on the other small tiller leaves of the same plant after 5 days. When the spikelets of wheat plants inoculated with BSMV-*TaPDS* showing symptoms of photobleaching were completely extracted (2–3 days), *F. graminearum* was inoculated onto the spikelets of both the silenced and control plants, which were then sampled for observation. The primers used for qPCR analysis were designed by Primer Premier 5.0 software, and they are listed in Table S5. Tests were replicated three times.

### Statistical analysis

Data from three biological replicates were analyzed and plotted using Student’s t-test and expressed as mean ± standard deviation through GraphPad Prism software (version 9.5.0) (Swift 1997). Significant differences among the treatment groups were determined using one-way analysis of variance, and data were considered statistically significant at *p* < 0.05 or *p* < 0.01.

## Supplementary Information

Additional file 1: Figure S1. Microscopic observation of spikelet tissues of Xinong 511 and Aikang 58 spikes. Sections were stained by van gieson stain. The scale bar is located at the bottom right of the images. A, B, C, D: XN511; E, F, G, H: AK58. The lignified cell walls are red, while the cellulose cell walls are green. Figure S2. (A)Histogram of differentially expressed genes (DEGs); (B) Venn of all DEGs. Figure S3. Module MEred of weighted gene co-expression network analysis (WGCNA). (A) Correlation between gene significance and module nembership of tan module. (B) The GO analysis in module red. (C) The correlation network of module of tan. A gene network is constructed by WGCNA, in which each node represents a gene, and the connecting line (edge) between genes represents the co-expression correction. The size and color of each circle represent the number of edges. Figure S4. The sample correlation heatmap and GO analysis. (A). The sample correlation heatmap. (B). GO analysis plot between different treatment groups.

Additional file 2: Table S1. Summary of RNA-Seq reads mapping results. Table S2. GO enrichment of significant terms generated from differentially regulated genes. Table S3. DEGs were found in phenylpropanoid pathway. Table S4. Data of WGCNA analysis. Table S5. List of all primers used in this study. Table S6. Summary of RNA-Seq reads mapping results in population transcriptomics. Table S7. A FPKM value in population transcriptomics. Table S8. DEGs were found in population transcriptomics. Table S9. GO enrichment of significant terms generated from differentially regulated genes in population transcriptomics. Table S10. GO enrichment of significant terms generated from differentially expressed genes between XN511 and AK58 under mock-inoculated condition.

## Data Availability

The sequencing data from the transcriptome have been submitted to NCBI under study accession PRJNA1200567.

## Abbreviations

BSMV: Barley stripe mosaic virus
CAD: Cinnamoyl alcohol dehydrogenase
CCR: Cinnamoyl-Co A reductase
CYP: Cytochrome P450
DEGs: Differential expression genes
FDR: False discovery rate
FHB: *Fusarium* head blight
FPKM: Fragments per kilobase per million mapped reads
GO: Gene ontology
HMA: Heavy metal-associated
PAL: Phenylalanine ammonia lyase
PCA: Principal component analysis
PSS: Percentage of symptomatic spikelet
RILs: The recombinant inbred lines
SDW: Sterile distilled water
SFI: The single floret spikelet inoculation method
VIGS: Viral-induced gene silencing
WGCNA: Weighted gene co-expression network analysis
4CL: 4-Coumarate: CoA ligase
