# Supplementary material for: Molecular dissection of Xinong 511 spike rachis response to Fusarium head blight infection
Source: Stress Biol. 2025 Jul 23;5(1):48. doi: 10.1007/s44154-025-00240-x (PMC12287499; doi:10.1007/s44154-025-00240-x)
Supplement: Supplementary file 1 — Additional file 1: Figure S1. Microscopic observation of spikelet tissues of Xinong 511 and Aikang 58 spikes. Sections were stained by van gieson stain. The scale bar is located at the bottom right of the images. A, B, C, D: XN511; E, F, G, H: AK58. The lignified cell walls are red, while the cellulose cell walls are green. Figure S2. (A)Histogram of differentially expressed genes (DEGs); (B) Venn of all DEGs. Figure S3. Module MEred of weighted gene co-expression network analysis (WGCNA). (A) Correlation between gene significance and module nembership of tan module. (B) The GO analysis in module red. (C) The correlation network of module of tan. A gene network is constructed by WGCNA, in which each node represents a gene, and the connecting line (edge) between genes represents the co-expression correction. The size and color of each circle represent the number of edges. Figure S4. The sample correlation heatmap and GO analysis. (A). The sample correlation heatmap. (B). GO analysis plot between different treatment groups. [file 44154_2025_240_MOESM1_ESM.docx]

**Molecular dissection of Xinong 511 Spike Rachis Response to Fusarium Head Blight Infection**

Xiaoying Yang^1^, Maoru Xu^1^, Guangyi Wang^1^, Xiaofang Cheng^1^, Zhengkai Feng^1^, Xiaoqi Zhao^1^, Tingdong Li^1,2^, Pingchuan Deng^1,2^, Changyou Wang^1,2^, Xinlun Liu^1,2^, Jixin Zhao^1,2^, Chunhuan Chen^1,2^, Wanquan Ji^1,2,^ *

^1^ College of Agronomy, Northwest A&F University, Yangling 712100, China

^2^ State Key Laboratory of Crop Stress Biology for Arid Areas, Yangling 712100, China

* For correspondence (e-mail [jiwanquan2008@126.com](mailto:jiwanquan2008@126.com)).

**Supplementary figures**

**Fig. S1** A schematic illustration of the anatomy of the wheat ear and the vascular connections between neighbouring spikelets. Blue dotted lines indicate plant vasculature; red arrows represent the overall direction of fungal infection; red numbers denote the stages of fungal infection described as described previously (Brown *et al.*, 2010); red dotted lines mark the rachis nodes; red circles indicate the rachilla.

**Fig. S2** (A)Histogram of differentially expressed genes (DEGs); (B) Venn of all DEGs.

**Fig. S3** Module MEred of weighted gene co-expression network analysis (WGCNA). (A) Correlation between gene significance and module nembership of tan module. (B) The GO analysis in module red. (C) The correlation network of module of tan. A gene network is constructed by WGCNA, in which each node represents a gene, and the connecting line (edge) between genes represents the co-expression correction. The size and color of each circle represent the number of edges.

**Fig. S4** The sample correlation heatmap and GO analysis. (A). The sample correlation heatmap. (B). GO analysis plot between different treatment groups.

Fig. S1 A schematic illustration of the anatomy of the wheat ear and the vascular connections between neighbouring spikelets. Blue dotted lines indicate plant vasculature; red arrows represent the overall direction of fungal infection; red numbers denote the stages of fungal infection described as described previously (Brown *et al.*, 2010); red dotted lines mark the rachis nodes; red circles indicate the rachilla.


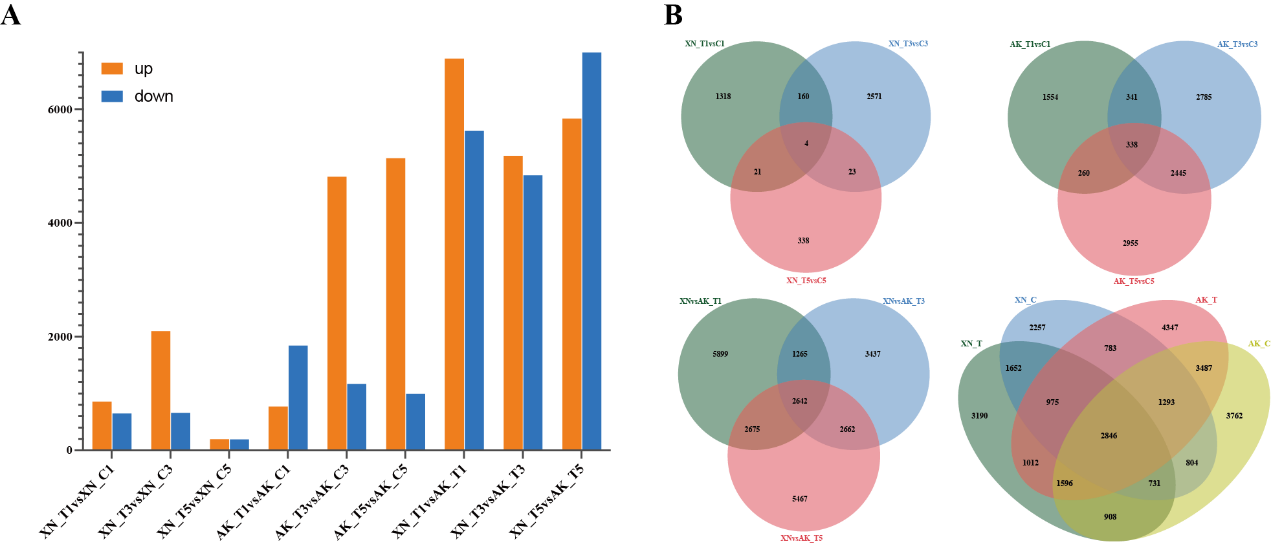


**Fig. S2** (A)Histogram of differentially expressed genes (DEGs); (B) Venn of all DEGs.


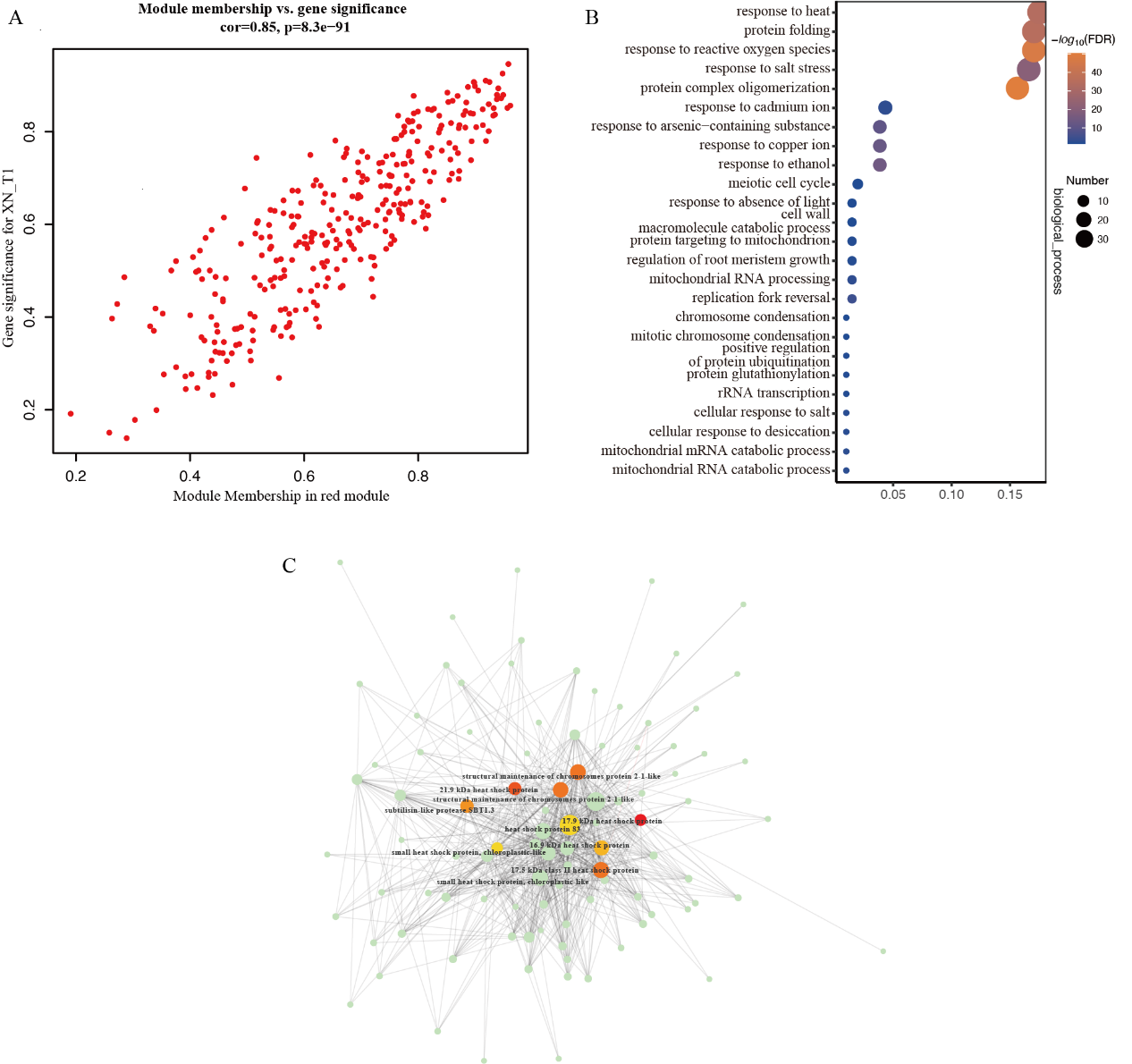


**Fig. S3** Module MEred of weighted gene co-expression network analysis (WGCNA). (A) Correlation between gene significance and module nembership of tan module. (B) The GO analysis in module red. (C) The correlation network of module of tan. A gene network is constructed by WGCNA, in which each node represents a gene, and the connecting line (edge) between genes represents the co-expression correction. The size and color of each circle represent the number of edges.


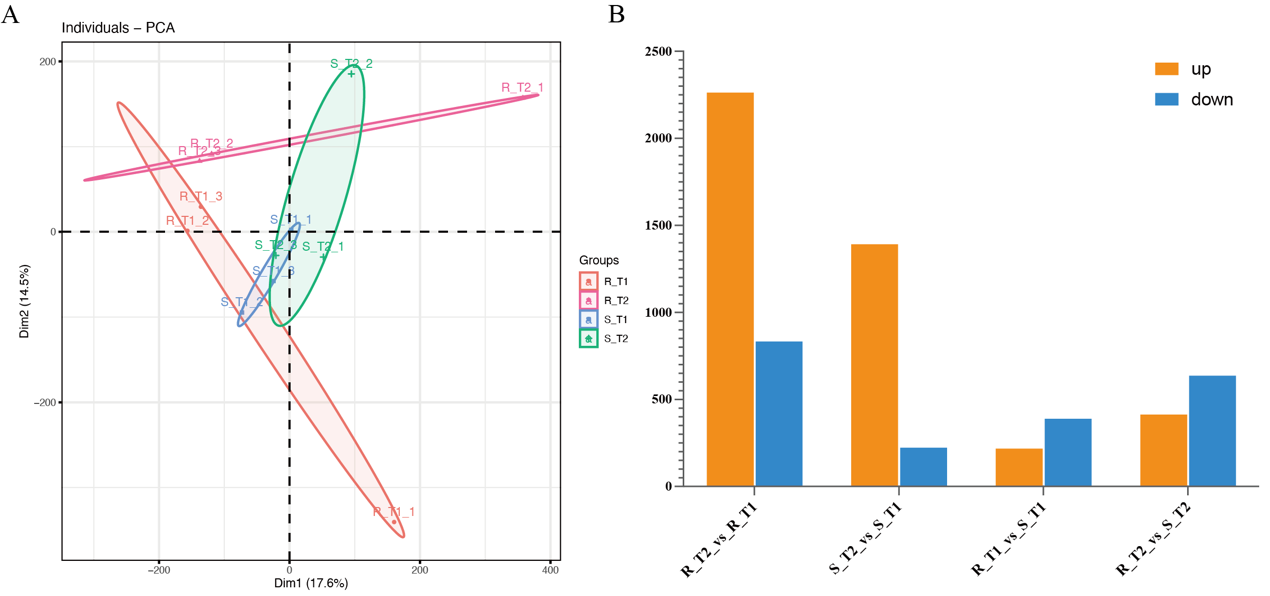


**Fig. S4** The sample correlation heatmap and GO analysis. (A). The sample correlation heatmap. (B). GO analysis plot between different treatment groups.

Brown NA, Urban M, van de Meene AM et al (2010) The infection biology of Fusarium graminearum: defining the pathways of spikelet to spikelet colonisation in wheat ears. Fungal Biol 114(7), 555-571. https://doi.org/10.1016/j.funbio.2010.04.006
